# Association between systemic immune-inflammation index and trimethylamine N-oxide levels in peripheral blood and osteoporosis in overweight and obese patients

**DOI:** 10.3389/fendo.2025.1539594

**Published:** 2025-02-13

**Authors:** Lingling Li, Jinyang An, Jia Bai, Yangyang Zhang, Xinsai Li, Haihong Lv

**Affiliations:** ^1^ The First School of Clinical Medicine, Lanzhou University, Lanzhou, China; ^2^ Department of Endocrinology, The First Hospital of Lanzhou University, Lanzhou, China

**Keywords:** overweight and obesity, systemic immune-inflammation index, trimethylamine N-oxide, bone mineral density, osteoporosis, visceral fat area

## Abstract

**Background:**

The intricate relationship between systemic immune-inflammation index (SII) and trimethylamine N-oxide (TMAO) in the peripheral blood and osteoporosis (OP) remains unclear. This study aims to investigate variations in the levels of SII and TMAO in the peripheral blood of overweight and obese patients, and examine the associations between these markers, bone mineral density (BMD), and the occurrence of osteoporotic fractures.

**Methods:**

The study enrolled 765 patients aged ≥ 50 years with BMI ≥ 24 kg/m², dividing them into two groups based on visceral fat area (VFA): <100 cm² and ≥100 cm². A corrected regression model analyzed the association of SII, TMAO, BMD, and osteoporotic fractures incidence in patients with central obesity. Receiver operator characteristic (ROC) curves assessed the predictive ability of SII and TMAO for OP screening.

**Results:**

Baseline data showed that patients with VFA ≥ 100 cm² had lower whole body (WB) and lumbar spine (LS) BMD, but higher SII and TMAO levels compared to those with VFA < 100 cm² (p < 0.05). Particularly in the group with VFA ≥ 100 cm^2^, there was an upward trend in SII and TMAO as bone mass decreased. Regression analysis found SII and TMAO negatively correlated with WB, LS, and femoral neck (FN) BMD, and positively correlated with osteoporotic fractures incidence (p < 0.05). Both were independent risk factors for OP, with combined SII and TMAO detection showing high diagnostic efficacy (sensitivity 94.7%, specificity 96.5%).

**Conclusion:**

In overweight and obese patients, particularly those with a VFA ≥ 100 cm², peripheral blood SII and TMAO levels may serve as valuable biomarkers for the early diagnosis of OP, offering potential clinical utility in identifying high-risk individuals.

## 1 Introduction

Obesity and osteoporosis (OP) are increasingly prevalent chronic health conditions, with a growing global incidence rate (1). Previously, it was believed that obesity conferred protection to bones due to the increased mechanical load from body weight, leading to higher bone mineral density (BMD) and decreased risk of OP (2, 3). However, recent studies have demonstrated that adipose tissue, particularly visceral adipose tissue, can exert additional detrimental effects on skeletal health (4). It is worth noting that some overweight individuals may also have central fat accumulation. Therefore, there is a need to explore new indicators for OP and osteoporotic fractures (OPF) in overweight and obese individuals, providing new insights for managing the risk of OP in patients with central obesity.

Overweight and obesity can lead to a state of chronic low-grade inflammation in the body (5). The systemic immune-inflammation index (SII) is a new indicator based on lymphocyte, neutrophil, and platelet counts that offers the advantages of simplicity, non-invasiveness, and cost-effectiveness. It comprehensively reflects the overall immune and inflammatory state of the body and is less influenced by short-term physiological fluctuations compared to single inflammatory markers such as CRP, IL-1, and TNF-α (6). Previous studies have indicated that SII has potential application value in risk assessment and prognosis (7, 8). A cross-sectional survey study involving American adults found a positive correlation between body mass index (BMI) and SII. Inflammation also plays a crucial role in bone remodeling, as OP has been closely linked to systemic immune and inflammatory states (9), potentially influenced by direct or indirect effects of immune cells on physiological processes within bone cells (10, 11). Tang et al. discovered that elevated levels of SII could potentially increase the risk for OP in postmenopausal women aged ≥ 50 years. This suggests its potential use in predicting the occurrence risk of low BMD or OP among this demographic group (6), which was supported by similar conclusions reached by Zhang (12) and Du et al (13). However, these studies are all based on the BMD of postmenopausal women, and there is a paucity of research on OP in overweight and obese patients.

The process of overweight and obesity can also trigger chronic inflammation and metabolic disorders in the intestines, with the composition and activity of the gut microbiome playing a pivotal role in initiating adipose tissue inflammation (14). Trimethylamine N-oxide (TMAO), which is a dietary choline and other trimethylamine-containing nutrient-dependent metabolic product of the gut microbiome, has been validated as an indicator of dysregulated gut microbiome metabolism in obese patients (15). In recent years, there has been significant attention on exploring the relationship between bone metabolism and gut microbial metabolism. Several studies have demonstrated that the gut microbial metabolites can regulate bone metabolism by influencing host metabolism, immune function, and hormone secretion through what is known as the “bone-gut” axis (16, 17). Our previous research revealed a significant linear correlation between TMAO levels and BMD in T2DM patients (18). However, there has been limited investigation into the relationship between TMAO and OP in overweight and obese patients.

Abnormal changes in the levels of SII and TMAO have been observed in individuals with overweight and obesity. However, there is limited research on the association between these factors and BMD or the risk of OPF in overweight and obese patients. Considering that SII and TMAO may serve as a link between obesity and OP through systemic and intestinal inflammation, this study seeks to investigate variations in these biomarkers among overweight and obese individuals, as well as their correlation with BMD and OPF. This study provides new targets for identifying clinical indicators of OP in overweight and obese populations.

## 2 Materials and methods

### 2.1 Study population

According to the inclusion and exclusion criteria, 765 patients aged ≥ 50 years and with a BMI ≥ 24 kg/m^2^ (19) were selected from the endocrinology outpatient clinic and inpatient department of Lanzhou University First Hospital between May 2022 and May 2024 (475 male and 290 female). Based on the criteria for visceral adiposity (20), the subjects were categorized into two groups: VFA < 100 cm^2^ and VFA ≥ 100 cm^2^. All investigations followed the ethical principles of the Helsinki Declaration, and the study was approved by the ethics committee of the First Hospital of Lanzhou University.

### 2.2 Inclusion and exclusion criteria

Inclusion: (1) aged ≥50 years, and female were natural menopause; (2) BMI ≥ 24kg/m^2^. Exclusion: (1) premenopausal women; (2) patients with diseases affecting bone metabolism, such as diabetes, Cushing’s syndrome, thyroid or parathyroid diseases, and rheumatoid arthritis; (3) patients who have recently been prescribed medications that impact bone metabolism, including anti-epilepsy drugs, antidepressants, thiazolidinediones, glucocorticoids, calcium, calcitriol, calcitonin, bisphosphonates, thyroid hormones and diuretics. (4) patients with recent acute or chronic infections; (5) patients diagnosed with solid or hematological malignancies; (6) patients who have received antibiotic or probiotic therapies within the past 3 months; (7) patients exhibiting significant liver or kidney dysfunction; and (8) patients with incomplete clinical data.

### 2.3 Research methods

#### 2.3.1 General information collection

Thoroughly obtain the patient’s medical history, including age, gender, medication use, smoking and drinking habits, and history of OPF. Fracture history was obtained through reviewing medical records or from patients who provided their own medical and imaging documentation. OPF (also known as fragility fractures) are fractures that occur due to minor trauma (such as falling from a standing height or lower) (21), and the timeframe for fractures is 0-14 years in this study. Measure the patient’s height, weight, systolic blood pressure (SBP), diastolic blood pressure (DBP), and record the average values. Calculate the body mass index (BMI) using the formula BMI = weight/height^2^ (kg/m^2^).

#### 2.3.2 Laboratory index determination

The patients underwent a 10-hour fasting period prior to morning blood sample collection. Total cholesterol (TC), triglyceride (TG), high-density lipoprotein cholesterol (HDL-C), low-density lipoprotein cholesterol (LDL-C), aspartate aminotransferase (AST), alanine aminotransferase (ALT), blood calcium (Ca), blood phosphorus (P) and alkaline phosphatase (ALP) were measured by automatic biochemical analyzer (AU5800 automatic biochemical analyzer, Beckman Coulter Diagnostics, California, USA). The levels of 25 hydroxyvitamin D (25(OH)D), type I collagen amino terminal peptide (PINP), and type I collagen carboxy-terminal peptide β special sequence (β-CTX), parathyroid hormone (PTH) and osteocalcin (OC) were measured by electrochemiluminescence immunoassay (Cobas e 801 immunoassay analyzer, Roche Diagnostics, Basel, Switzerland). Neutrophil count, lymphocyte count, and platelet count were measured by an automatic blood cell analyzer (Horiba Pentra XL 80, Horiba Medical, Kyoto, Japan). SII was calculated as follows: platelet count × neutrophil count/lymphocyte count (22).

#### 2.3.3 VFA measurement

The VFA was assessed with an dual-phase scanning HDS-2000 device (Omron, Japan). All subjects underwent a minimum 10-hour fast and were required to void their bowels and bladder prior to measurement. Subjects were positioned in the supine posture, instructed to remain still, and then VFA was measured by a trained operator following the HDS-2000 operation manual.

#### 2.3.4 Serum TMAO assay

The fasting blood samples were collected from patients following a 10-hour fast, and subsequently centrifuged at 3000 rpm for 10 minutes at 4°C and then stored at −80°C. TMAO levels in the serum were determined using stable isotope dilution high-performance liquid chromatography-mass spectrometry (HPLC-MS/MS) (Agilent Technologies, USA). The serum sample (60 μL) was mixed with 100 μL of internal standard (d9-TMAO, 10 μmol/L). The proteins in the sample were precipitated by vortexing for 1 minute, followed by centrifugation at 13200rpm, 4°C for 15 minutes to recover the supernatant. 5 μL supernatant was injected into SiO2 column (2.1 mm × 100 mm, 5 μm) for analysis. An isocratic elution was performed using 30% solution A (10 mmol/L ammonium formate, pH 3.0) and 70% solution B (acetonitrile) at a flow rate of 0.4 mL/min. The column temperature was set at 30°C. The sample was analyzed using positive multiple reaction monitoring (MRM) mode. The serum TMAO concentration was determined by comparing the results with a standard calibration curve.

#### 2.3.5 BMD measurement and OP diagnosis

WB BMD, LS BMD and FN BMD were measured by a dual-energy X-ray bone densitometer (Lunar iDXA, GE Healthcare), and T scores were automatically analyzed by the software. Prior to each scan, the DXA scanner must be calibrated in accordance with the manufacturer’s recommendations. The diagnostic criteria are as follows: Postmenopausal women and men over 50 years old can be diagnosed with OP if they meet any of the following criteria: (1) T-score −2.5 or below in the lumbar spine, femoral neck, total proximal femur, or 1/3 radius; (2) Low-trauma spine or hip fracture (regardless of BMD); (3) T-score between −1.0 and −2.5 and a fragility fracture of proximal humerus, pelvis, or distal forearm. T-score ≥ -1.0 indicates normal bone mass, and T score between -2.5 and -1.0 indicates osteopenia (23).

### 2.4 Statistical analysis

The data were analyzed using SPSS 26.0 software. Quantitative data that followed a normal distribution were expressed as mean ± standard deviation (X ± s), and comparisons between the two groups were made using t-test. Quantitative data that did not follow a normal distribution were expressed as median and interquartile range [M(P25, P75)], and comparisons between the three groups were made using Kruskal-Wallis H test, and comparisons between pairs were made using Wilcoxon rank sum test. Categorical data were expressed as frequency and percentage [n (%)], and comparisons between groups were made using chi-square test. A multiple linear regression model and a binary Logistic regression model were used to analyze the relationship between SII, TMAO, and BMD, fracture incidence in patients with central obesity. A multivariate Logistic regression model was used to analyze the influencing factors of central obesity-related OP. The predictive ability of SII and TMAO for central obesity-related OP was evaluated using the receiver operating characteristic curve (ROC). p < 0.05 was considered statistically significant.

## 3 Results

### 3.1 General information of subjects and levels of indicators

**Figure 1:**
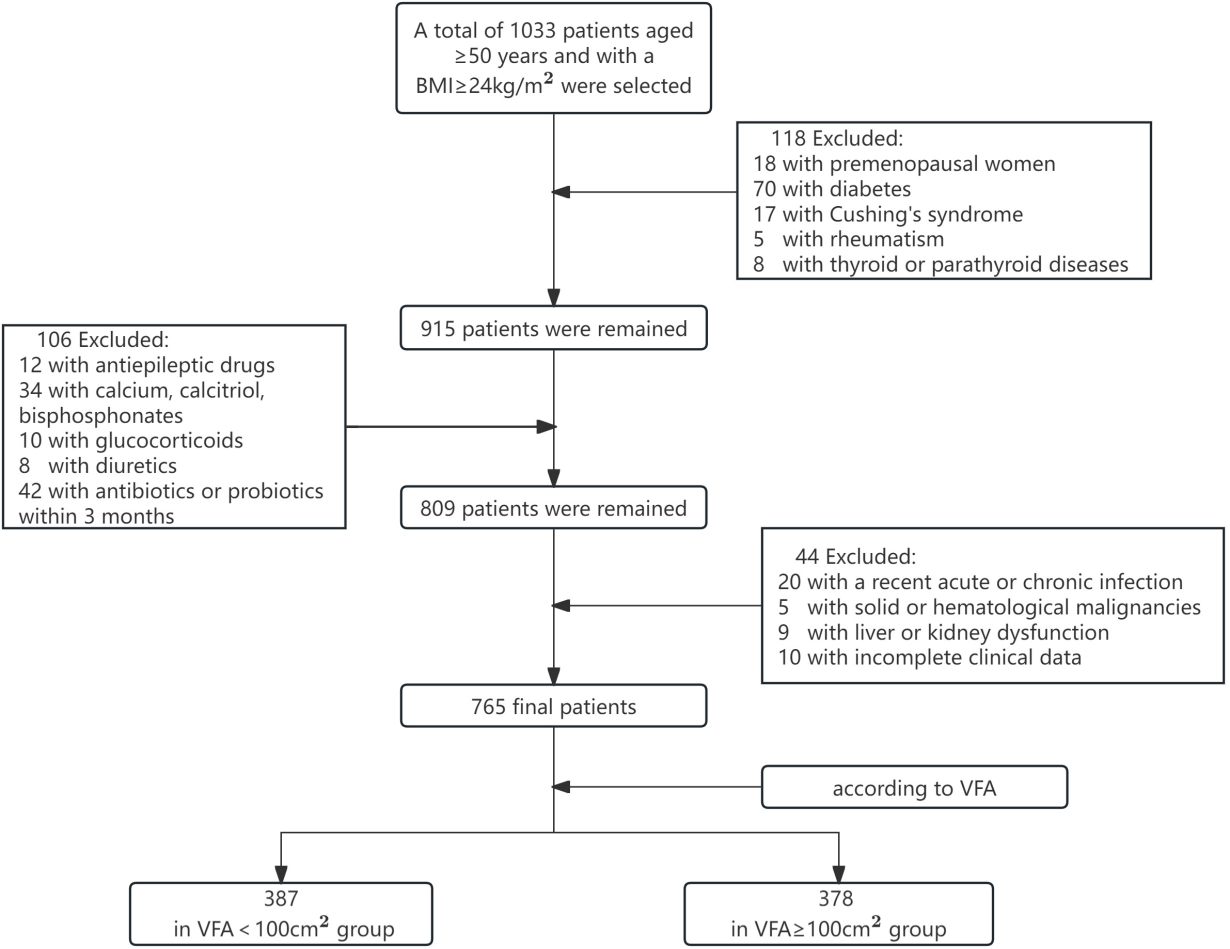
The flowchart of the screening process. VFA, visceral fat area.

The flowchart of the screening process is depicted in **Figure 1**. This study included a total of 765 patients with a BMI ≥ 24kg/m^2^, with an average age of 62.4 ± 7.8 years, including 475 males and 290 females with natural menopause. As shown in **Table 1**, subjects were divided into two groups according to the VFA, of which 387 were VFA < 100cm^2^ and 378 were VFA ≥ 100cm^2^. There were significant differences in ALT, TC, LDL-C, SII, TMAO, WB BMD, and LS BMD between the two groups (*p < 0.05*). Compared with the VFA < 100 cm^2^ group, patients with VFA ≥ 100 cm^2^ had higher levels of TC, LDL-C, SII, TMAO, and lower levels of ALT, WB BMD, and LS BMD. There were no significant differences in age, BMI, smoking rate, alcohol consumption rate, SBP, DBP, AST, TG, HDL-C, Ca, P, ALP, PTH, 25(OH)D, OC, PINP, B-CTX, and FN BMD between two groups (*p* > 0.05).

**Table 1:** Clinical baseline data of patients with different fat distribution.

	All (n=765)	VFA<100cm^2^ (n= 387)	VFA≥100 cm^2^ (n=378)	*p* Value
Age (y)	62.4 ± 7.8	62.1 ± 7.7	62.8 ± 7.9	0.220
Gender (male/female)	475/290	250/137	225/153	0.148
BMI (kg/m^2^)	25.6 (24.6-27.0)	25.7 (24.6-27.0)	25.5 (24.6-27.1)	0.670
Smoking (%)	168 (22%)	90 (23.3%)	78 (20.9%)	0.381
Drinking (%)	142 (18.6%)	78 (20.2%)	64 (16.9%)	0.252
SBP (mmHg)	137.2 ± 19.7	137.0 ± 18.7	137.5 ± 20.7	0.706
DBP (mmHg)	80.4 ± 11.2	80.2 ± 10.8	80.5 ± 11.5	0.699
AST (U/L)	19.0 (16.0-24.0)	19.0 (16.0-25.0)	18.0 (15.0-23.3)	0.289
ALT (U/L)	19.0 (14.0-28.0)	19.0 (15.0-28.0)	18.0 (14.0-27.0)	0.044*
TC (mmol/L)	4.4 (3.7-5.1)	4.3 (3.6-5.0)	4.5 (3.8-5.2)	0.003*
TG (mmol/L)	1.5 (1.1-2.1)	1.5 (1.1-2.0)	1.5 (1.1-2.1)	0.806
HDL-C (mmol/L)	1.0 (0.9-1.2)	1.0 (0.9-1.2)	1.0 (0.90-1.2)	0.091
LDL -C (mmol/L)	2.7 (2.1-3.3)	2.7 (2.1-3.2)	2.8 (2.2-3.3)	0.043*
Neutrophil (10^9^/L)	3.3 (2.6-4.3)	3.3 (2.6-4.3)	3.3 (2.6-4.2)	0.804
Platelet (10^9^/L)	187.0 (149.0-226.5)	182.0 (146.0-221.0)	191.0 (150.8-233.0)	0.031*
Lymphocyte (10^9^/L)	1.9 (1.5-2.3)	1.8 (1.5-2.2)	1.9 (1.5-2.4)	0.292
SII	324.2 (231.1-465.4)	311.1 (213.7-443.3)	335.3 (243.6-508.8)	0.009*
TMAO (μmol/L)	4.4 (3.8-4.9)	4.1 (3.6-4.5)	4.9 (4.1-5.8)	<0.001*
Ca (mmol/L)	2.2 (2.1-2.2)	2.2 (2.1-2.3)	2.2 (2.1-2.2)	0.830
P (mmol/L)	1.2 (1.1-1.3)	1.2 (1.1-1.3)	1.1 (1.1-1.3)	0.171
ALP (U/L)	69.3 (53.4-86.4)	67.5 (52.9-85.4)	70.0 (54.5-88.1)	0.210
PTH (pg/mL)	35.2 (28.5-46.9)	35.3 (27.7-46.9)	35.2 (28.8-46.8)	0.790
25 (OH)D (ng/mL)	13.2 (9.4-17.4)	13.1 (9.9-17.0)	13.2 (8.9-17.8)	0.549
OC (ng/mL)	13.2 (9.8-16.7)	12.9 (9.6-16.6)	13.3 (10.2-17.0)	0.169
PINP (ng/mL)	36.2 (28.6-47.2)	38.2 (28.2-50.6)	35.3 (28.1-44.1)	0.053
B-CTX (pg/mL)	424.0 (311.5-533.5)	423.5 (318.0-491.8)	429.0 (301.0-593.0)	0.138
WB BMD (g/cm^2^)	1.083 (0.952-1.188)	1.102 (0.983-1.195)	1.059 (0.925-1.182)	0.001*
LS BMD (g/cm^2^)	1.080 (0.950-1.220)	1.100 (0.978-1.230)	1.060 (0.920-1.203)	0.001*
FN BMD (g/cm^2^)	0.875 (0.757-1.009)	0.876 (0.719-1.012)	0.874 (0.769-1.006)	0.168

*p < 0.05, statistically significant difference.

VFA, visceral fat area; BMI, body mass index; SBP, systolic blood pressure; DBP, diastolic blood pressure; AST, aspartate aminotransferase; ALT, alanine aminotransferase; TC, total cholesterol; TG, triglyceride; HDL-C, high-density lipoprotein; LDL-C, low-density lipoprotein; SII, systemic immune-inflammation index; TMAO, trimethylamine N-oxide; Ca, calcium; P, phosphorus; ALP, alkaline phosphatase; PTH, parathyroid hormone; 25 (OH)D, 25-hydroxyvitamin D; OC, osteocalcin; PINP, procollagen type I N-propeptide; β-CTx, beta-isomer of the C-terminal telopeptide of type I collagen; WB, whole body; BMD, bone mineral density; LS, lumbar spine; FN, femoral neck.

### 3.2 Comparison of SII and TMAO levels in overweight and obese patients with different bone mass

**Figure 2:**
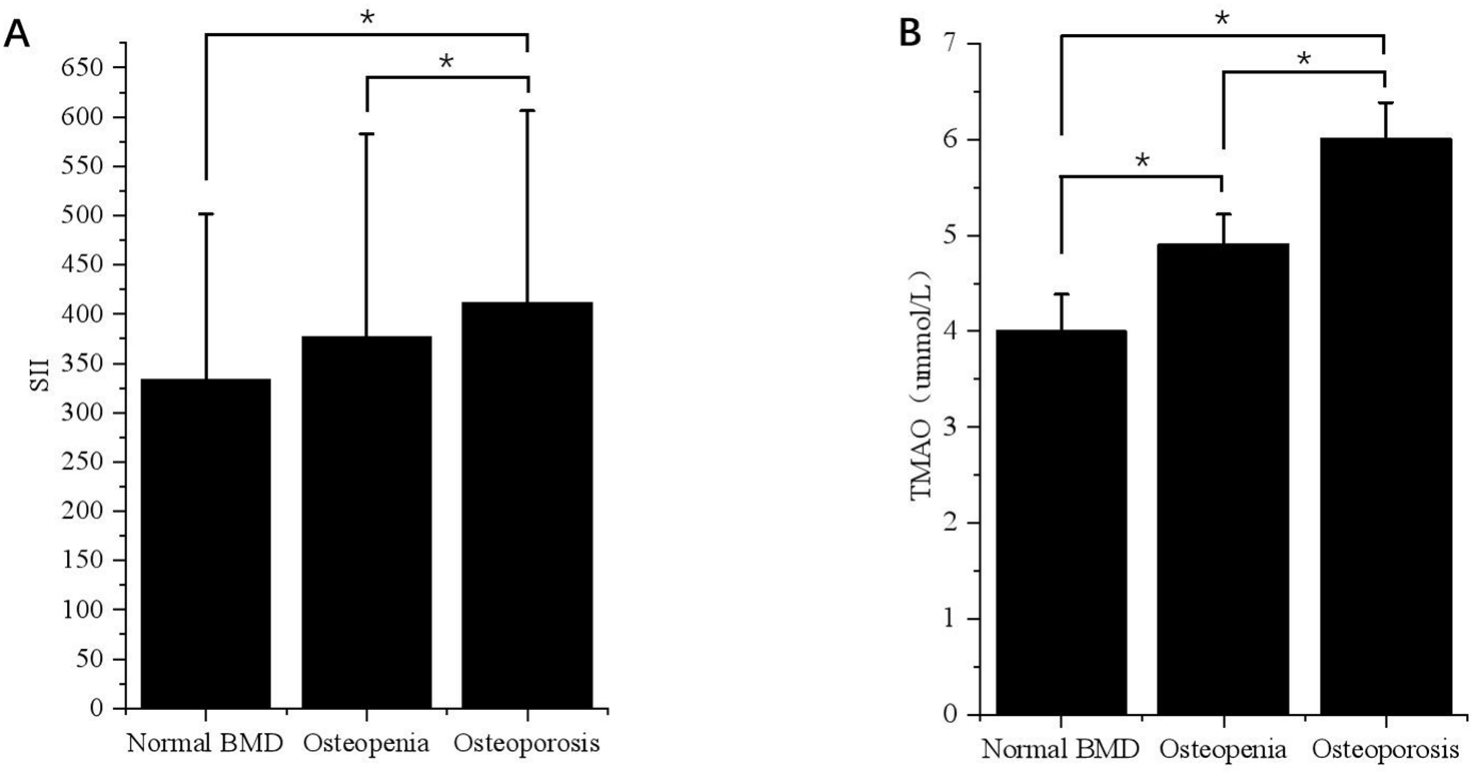
Differences in SII and TMAO levels in patients with VFA ≥ 100cm^2^ under different bone mass. **(A)** Differences in SII levels in VFA ≥ 100cm² patients with different bone mass. *p < 0.05. **(B)** Differences in TMAO levels in patients with VFA ≥ 100cm^2^ under different bone mass. *p < 0.05.

According to the BMD results, the subjects were divided into normal bone mass group, osteopenia group, and OP group, and the levels of SII and TMAO in overweight and obese patients with different bone mass were analyzed. As shown in **Table 2**, the average SII levels for all subjects in the three groups were: 296.75 (208.47-411.45), 327.66 (234.21-459.89), 376.89 (261.97-614.10), compared with the first two groups, the level of SII in OP group was the highest (*p* < 0.001). SII was also higher in the osteopenia group than in the normal bone mass group (*p* < 0.001). The results in male patients were consistent with the general population, but in female patients, the level of SII in the OP group was higher than that in the normal bone mass group (*p* < 0.05), but the difference was not statistically significant compared with the osteopenia group (*p* > 0.05). Subgroup analysis showed that there was no statistically significant difference in SII levels among different bone mass patients in the VFA < 100cm^2^ group, and no significant difference between male and female patients (*p* > 0.05). In the total population of the VFA ≥ 100cm^2^ group, the level of SII in the OP group was higher than that in the first two groups (*p* < 0.001), and there was no statistically significant difference between the osteopenia group and the normal bone mass group (*p* > 0.05), but it also showed an increasing trend with the decrease of bone mass (**Figure 2A**). The difference was also significant in males; In female patients, the level of SII in the OP group was higher than that in the normal group (*p* < 0.05), but the difference was not statistically significant compared with the osteopenia group (*p* > 0.05).

**Table 2:** Comparison of SII and TMAO levels in patients with normal bone mass, osteopenia, and OP.

		Normal (n= 327)	Osteopenia (n=229)	OP (n= 209)	p Value
SII	All				
	Total	296.75 (208.47-411.45)	327.66 (234.21-459.89)^a^	376.89 (261.97-614.10)^ab^	<0.001*
	Males	296.80 (210.75-397.13)	364.70 (261.49-457.75)^a^	392.73 (257.72-704.30)^ab^	<0.001*
	Females	295.40 (195.23-445.20)	318.16 (220.12-487.88)	369.85 (267.01-585.41)^a^	0.019*
	VFA<100cm^2^				
	Total	296.50 (204.40-416.40)	330.20 (252.50-450.60)	335.00 (196.20-685.90)	0.835
	Males	313.39 (224.68-453.07)	298.80 (206.46-430.39)	324.56 (219.25-365.97)	0.840
	Females	296.91 (208.47-490.65)	314.07 (222.00-451.52)	312.86 (187.13-512.19)	0.906
	VFA≥100 cm^2^				
	Total	296.99 (214.77-399.98)	312.14 (224.60-487.88)	403.85 (283.39-660.78)^ab^	<0.001*
	Males	298.85 (223.52-394.23)	309.71 (225.09-459.89)	419.74 (280.28-704.30)^ab^	<0.001*
	Females	278.09 (190.95-419.27)	326.40 (216.40-492.52)	377.37 (286.63-640.18)^a^	0.010*
TMAO	All				
	Total	4.00 (3.66-4.36)	4.58 (3.91-4.89)^a^	5.79 (4.51-6.15)^ab^	<0.001*
	Males	4.00 (3.64-4.36)	4.57 (3.75-4.89)^a^	5.76 (4.37-6.19)^ab^	<0.001*
	Females	4.00 (3.74-4.34)	4.59 (4.02-4.90)^a^	5.83 (4.78-6.15)^ab^	<0.001*
	VFA<100cm^2^				
	Total	4.00 (3.66-4.35)	4.10 (3.45-4.60)	4.22 (3.92-4.63)	0.065
	Males	4.00 (3.64-4.35)	4.05 (3.44-4.58)	4.19 (3.83-4.60)	0.261
	Females	4.00 (3.69-4.32)	4.18 (3.46-4.63)	4.24 (4.05-4.75)	0.100
	VFA≥100 cm^2^				
	Total	4.00 (3.67-4.36)	4.89 (4.64-5.17)^a^	5.94 (5.73-6.26)^ab^	<0.001*
	Males	3.95 (3.64-4.36)	4.89 (4.64-5.13)^a^	5.90 (5.66-6.29)^ab^	<0.001*
	Females	4.10 (3.77-4.38)	4.90 (4.63-5.18)^a^	6.00 (5.78-6.24)^ab^	<0.001*

*p < 0.05, statistically significant difference.

‘a’ means that compared with the Normal group, *p* < 0.05; ‘b’ indicated that compared with Osteopenia, *p* < 0.05;

The average TMAO levels in the three groups were as follows: 4.00 (3.66-4.36), 4.58 (3.91-4.89), and 5.79 (4.51-6.15). Among the total population, OP patients had the highest TMAO level, followed by the osteopenia group, and the normal bone mass group had the lowest level of TMAO. This difference was significant for both males and females (*p* < 0.05). In the subgroup with VFA < 100cm^2^, there was no statistically significant difference in TMAO levels among patients with normal bone mass, osteopenia, and OP. The results of the VFA ≥ 100cm^2^ group were consistent with those of the general population, showing that TMAO gradually increased with decreasing bone mass (*p* < 0.05) (**Figure 2B**).

### 3.3 Relationship between SII, TMAO and BMD, OPF in patients with central obesity

**Figure 3:**
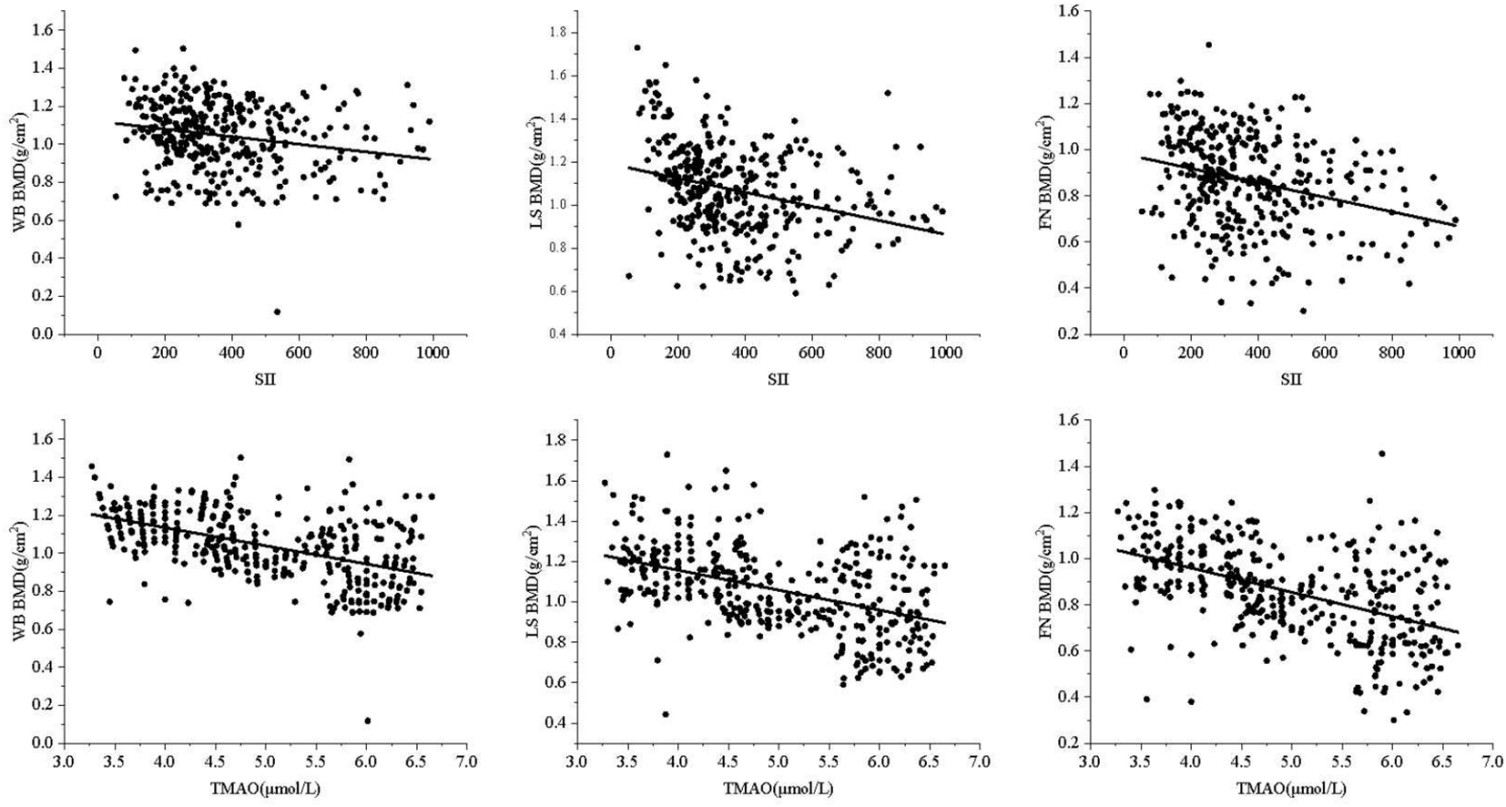
Correlation between SII, TMAO, and BMD.

Central obesity patients with VFA of ≥ 100 cm² were selected for the follow-up study. The scatter plot reveals a downward trend in WB BMD, LS BMD, and FN BMD as the levels of SII and TMAO increase (**Figure 3**). Subsequently, a linear regression model was used to further analyze the relationship between SII, TMAO and BMD in central obesity patients (**Table 3**). Model 1 is a basic model that includes SII or TMAO as a single independent variable to preliminarily explore their relationship with BMD and the occurrence of fragility fractures in patients with central obesity. Based on Model 1, Model 2 incorporates age, sex, and BMI as covariates according to literature reports (24), as these are recognized as key factors closely associated with OP. Model 3 further builds on Model 2 by adding variables that showed significant differences between the two groups in **Table 1** (P < 0.05), including TC, ALT, and LDL-C, to control for potential group differences. On the basis of the covariates in Model 3, Model 4 introduces biochemical markers closely related to OP—25(OH)D, PINP, and β-CTX—which reflect different aspects of bone metabolism. These factors are controlled to comprehensively assess the independent effects of SII and TMAO on OP.

**Table 3:** Model regression analysis of SII, TMAO, and BMD in central obesity patients.

		WB BMD		LS BMD		FN BMD	
		β	*p*	β	*p*	β	*p*
SII	Model 1	-0.161	0.002*	-0.242	<0.001*	-0.128	0.013*
	Model 2	-0.122	0.010*	-0.207	<0.001*	-0.107	0.035*
	Model 3	-0.125	0.009*	-0.205	<0.001*	-0.102	0.045*
	Model 4	-0.133	0.005*	-0.208	0.029*	-0.100	0.050
TMAO	Model 1	-0.500	<0.001*	-0.439	<0.001*	-0.492	<0.001*
	Model 2	-0.415	<0.001*	-0.360	<0.001*	-0.466	<0.001*
	Model 3	-0.417	0.013*	-0.359	<0.001*	-0.464	0.010*
	Model 4	-0.403	0.010*	-0.359	0.012*	-0.471	0.005*

*p < 0.05, statistically significant difference.

Model 1: not adjusted.

Model 2: adjusted for age, sex, BMI.

Model 3: adjusted for age, sex, BMI, TC, ALT, LDL-C.

Model 4: adjusted for age, sex, BMI, TC, ALT, LDL-C, 25(OH)D, PINP, B-CTX.

The results are shown in **Table 3**, where SII in patients with central obesity was negatively correlated with WB BMD, LS BMD, and FN BMD in Models 1, 2, and 3 (*p* < 0.05). In Model 4, after adjusting for age, sex, BMI, TC, ALT, LDL-C, 25(OH)D, PINP, and B-CTX levels, SII was still negatively correlated with WB BMD and LS BMD (*p* < 0.05). In Models 1-4, TMAO was negatively correlated with WB BMD, LS BMD, and FN BMD (*p* < 0.05). Using a binary logistic regression model (**Table 4**), it was found that the levels of SII and TMAO were positively correlated with the incidence of OPF in patients with central obesity Models 1-4(*p* < 0.05).

**Table 4:** Model regression analysis of SII, TMAO and fragility fracture in patients with central obesity.

		B	Wald	OR	OR (95% CI)	*p*
SII	Model 1	0.001	8.049	1.001	1.000-1.001	0.005*
	Model 2	0.001	6.025	1.001	1.000-1.001	0.014*
	Model 3	0.001	5.623	1.001	1.000-1.001	0.018*
	Model 4	0.001	6.662	1.001	1.000-1.001	0.010*
TMAO	Model 1	1.186	40.239	3.273	2.269-4.721	<0.001*
	Model 2	1.112	33.277	3.040	2.084-4.435	<0.001*
	Model 3	1.118	33.368	3.059	2.093-4.470	0.003*
	Model 4	1.110	32.257	3.035	2.069-4.451	0.011*

*p < 0.05, statistically significant difference.

Model 1: not adjusted.

Model 2: adjusted for age, sex, BMI.

Model 3: adjusted for age, sex, BMI, TC, ALT, LDL-C.

Model 4: adjusted for age, sex, BMI, TC, ALT, LDL-C, 25(OH)D, PINP, B-CTX.

**Figure 4:**
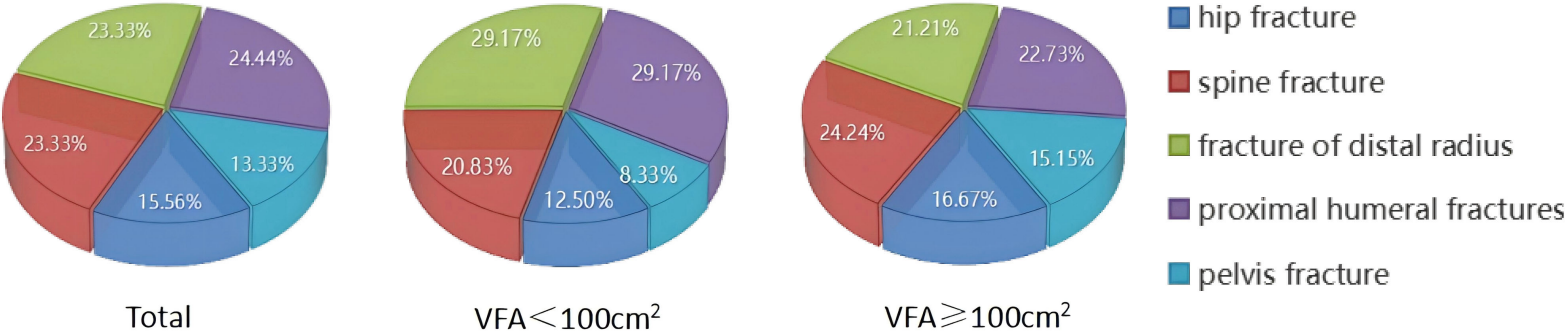
Proportion of individuals experiencing fractures in different parts.

The study quantified the number of fractures in various fracture sites (**Figure 4**). Out of a total group of 90 individuals with OPF, it was found that centrally obese patients with VFA ≥100cm^2^ accounted for a total of 66 fractures, including 11 hip fractures (16.67%), 16 spine fractures (24.24%), 14 distal radius fractures (21.21%), and 15 proximal humerus fractures (22.73%). Additionally, there were ten pelvis fractures, representing 15.15% of the total.

### 3.4 Multivariate logistic regression analysis of the influencing factors of central obesity patients with OP

To further explore the independent risk factors for OP in centrally obese patients, a multivariate logistic regression analysis was conducted with OP as the outcome variable. The results showed that after controlling for confounding factors, SII (OR = 1.005, 95% CI = 1.003-1.007, *p* < 0.001) and TMAO (OR = 2.733, 95% CI = 1.947-3.330, *p* < 0.001) were independent risk factors for OP in centrally obese patients (**Table 5**).

**Table 5:** Analysis of influencing factors in central obesity patients with osteoporosis.

	B	SE	Wald	OR	OR(95%CI)	*p*
age	0.061	0.033	3.359	1.063	0.996-1.134	0.067
Gender(female)	0.570	0.490	1.357	1.769	0.678-4.619	0.244
BMI	0.047	0.111	0.179	1.048	0.843-1.302	0.673
TC	0.202	0.210	0.923	1.224	0.810-1.849	0.337
ALT	-0.008	0.016	0.265	0.992	0.962-1.023	0.607
LDL -C	-0.162	0.293	0.305	0.851	0.479-1.510	0.581
PINP	-0.025	0.018	1.947	0.975	0.941-1.010	0.163
B-CTX	0.001	0.001	0.625	1.001	0.998-1.004	0.429
25(OH)D	-0.012	0.025	0.233	0.988	0.940-1.038	0.629
SII	0.005	0.001	31.870	1.005	1.003-1.007	<0.001*
TMAO	0.851	0.562	2.344	2.733	1.947-3.330	<0.001*

*p < 0.05, statistically significant difference.

### 3.5 ROC curve analysis of the predictive efficacy of SII and TMAO in central obesity patients with OP

**Figure 5:**
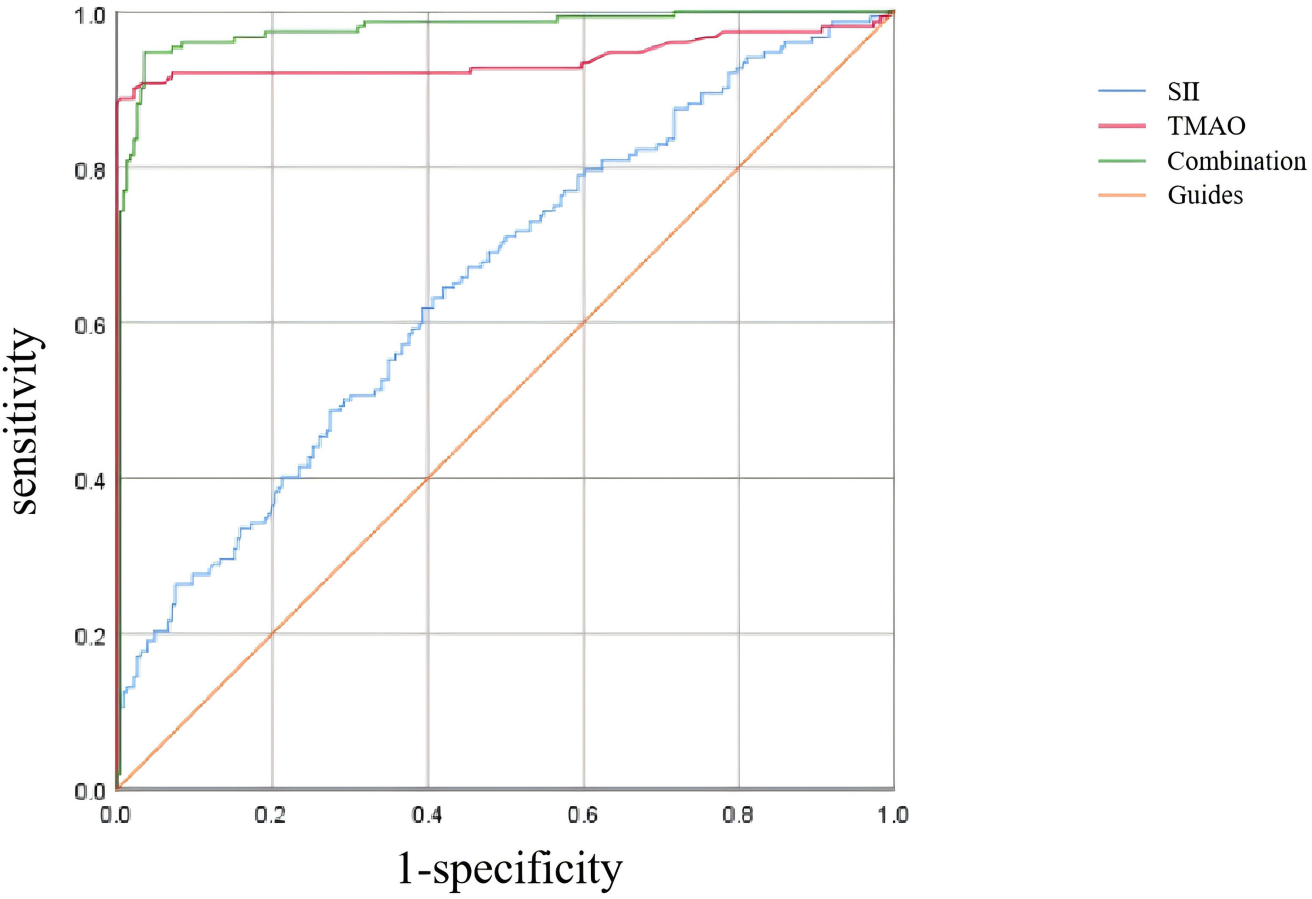
ROC curve analysis of SII and TMAO screening osteoporosis.

As shown in **Table 6** and **Figure 5**, according to the ROC curve analysis, the AUC of SII predicting OP in centrally obese patients was 0.652 (95% CI: 0.596-0.708, *p* < 0.001), with the maximum Jordan index of 0.225, and the corresponding optimal cut-off value of SII was 330.125. The sensitivity was 64.5%, and the specificity was 58.0%. The AUC of TMAO predicting OP in centrally obese patients was 0.939 (95% CI: 0.906-0.972, *p* < 0.001), with the maximum Jordan index of 0.884, and the corresponding optimal cut-off value of TMAO was 5.555. The sensitivity was 88.8%, and the specificity was 99.6%. The AUC of the combined use of SII and TMAO was the highest, at 0.976 (95% CI: 0.960-0.992, *p* < 0.001), with the sensitivity of 94.7% and the specificity of 96.5%. There were statistically significant differences in sensitivity (X^2^ = 37.144, *p* < 0.001) and specificity (X^2^ = 86.118, *p* < 0.001) among the three methods, and the sensitivity was highest for the combination of the two indicators.

**Table 6:** Predictive efficacy of SII and TMAO for osteoporosis in patients with central obesity (n=378).

	AUC	95% CI	Sensitivity (%)	Specificity (%)	optimal cut-off value
SII	0.652	0.596-0.708	64.5	58.0	330.125
TMAO	0.939	0.906-0.972	88.8	99.6	5.555
Combination	0.976	0.960-0.992	94.7	96.5	

AUC, Area Under the Curve.

## 4 Discussion

According to a study based on the China Health and Nutrition Survey, there has been a significant increase in the prevalence of general obesity and central obesity among adults in China over the past 20 years. The increase in overweight, whole-body obesity, and central obesity is more pronounced among men compared to women (25). Traditionally, it was believed that higher body weight increased mechanical load on bones, promoting bone formation and mineralization. Consequently, individuals with higher BMI typically had higher BMD and more soft tissue acting as cushioning which resulted in lower fracture risk for overweight and obese individuals. However recent studies have found that inflammation and other metabolic abnormalities associated with obesity may negatively impact bone health challenging traditional beliefs about the benefits of increasing fat mass for bones (4). The BMI solely provides a simplistic measurement of the correlation between weight and height, failing to capture the intricate distribution of various types of fat including visceral fat, subcutaneous fat, intramuscular fat, and marrow fat tissue. As individuals age, there may be no significant change in BMI; however, alterations occur in the distribution of adipose tissue with a decrease in subcutaneous fat and an increase in visceral fat, intramuscular fat, and marrow adiposity. Central obesity characterized by excessive accumulation of visceral adiposity adversely impacts skeletal health through mechanisms involving inflammation, oxidative stress, and hormonal dysregulation thereby contributing to decreased BMD and heightened risk for OP (5). The increased infiltration of fat into muscle leads to a decline in muscle strength, balance, and coordination, indirectly accelerating bone loss and increasing the risk of falls and fractures (26). Muscle wasting obesity (the coexistence of muscle wasting and fat accumulation) is closely associated with an elevated risk of falls and fractures, particularly when intramuscular fat further compromises muscle function (27), and visceral fat contributes to chronic inflammation that speeds up bone loss (5). Additionally, excessive marrow adipose tissue can alter the bone microenvironment and microstructure by replacing bone cells, resulting in decreased BMD (28). Moreover, BMI fails to differentiate between muscle mass and fat since muscles have higher density than fats. Therefore, individuals with high muscularity may exhibit a higher BMI but low body fat percentage. Muscle quality plays a protective role in skeletal health; thus strength training and increasing muscle mass can enhance skeletal health by improving BMD while reducing the incidence of OP (29). In conclusion, BMI inadequately reflects the risk of developing OP. For example someone with well-developed muscles may have a higher BMI but a lower body fat percentage. In this study, 765 patients were divided into two groups based on their BMI: the group with 24.0 ≤ BMI < 28kg/m^2^ and the group with BMI ≥ 28 kg/m^2^. No significant differences were observed between the two groups in terms of SII, TMAO, and BMD (data not shown), suggesting that studying the relationship between obesity and OP solely based on BMI is inadequate. A comprehensive consideration of various factors including fat distribution, mechanical loading, metabolic factors, and inflammatory status is necessary to understand the relationship between obesity and OP. Excessive visceral fat has been proven to have a more detrimental effect on skeletal health (30)and serves as a more accurate predictor of the risk of health problems than high BMI alone (31, 32).

VFA can quantify the accumulation of fat around vital internal organs such as the liver, intestines, and pancreas, making it a superior indicator for distinguishing visceral fat from subcutaneous fat in the abdomen compared to waist circumference and waist-to-hip ratio (33). Therefore, we selected VFA as the grouping criterion. The findings indicated that individuals with VFA ≥ 100 cm^2^ had lower WB BMD and LS BMD compared to those with VFA < 100 cm^2^, suggesting a potential link between visceral fat and bone loss, consistent with previous research. Kim et al. (34) demonstrated significantly lower FN BMD in patients with metabolic syndrome. Additionally, Bredella et al. (28) observed poorer bone microstructure and mechanical properties in men with high visceral fat content compared to those with low visceral fat content. A biopsy study has confirmed that postmenopausal women with central obesity exhibit lower bone quality and BMD (35). Meanwhile, this study also found that patients with VFA ≥ 100 cm^2^ exhibited elevated levels of SII and TMAO compared to those with VFA < 100 cm^2^, indicating that individuals with higher levels of visceral fat exhibit heightened inflammation within their bodies’ physiological systems. Visceral fat is metabolically active and can release more inflammatory factors, such as interleukin-6 and tumor necrosis factor-α. SII serves as a comprehensive indicator for systemic inflammation and immune response by integrating the counts of neutrophils, lymphocytes, and platelets. It is cost-effective and easily obtainable through routine blood tests, making it valuable for predicting and assessing prognosis in various diseases. The SII is closely associated with the secretion of inflammatory factors by adipose tissue, leading to alterations in neutrophil, lymphocyte, and platelet counts that influence the SII value. Elevated SII values typically indicate an upregulation of pro-inflammatory factors released by adipose tissue, suggesting an enhanced systemic inflammatory response (36). Obesity can also lead to chronic intestinal inflammation resulting in gut microbiome dysbiosis. Previous animal studies have demonstrated that inducing obesity in mice through a high-sugar, high-fat diet led to increased plasma TMAO levels along with heightened expression of tumor necrosis factor-α and interleukin-1β while decreasing the expression of anti-inflammatory cytokine interleukin-10 (37).

In order to investigate the association between SII, TMAO, and BMD in overweight and obese patients, we categorized the patients into groups based on their bone mass (normal, osteopenia, and OP) and analyzed the levels of SII and TMAO. The results revealed that both total population and gender-specific analyses showed higher levels of SII and TMAO in overweight and obese patients with OP compared to those with normal bone mass or osteopenia. Furthermore, there was a trend of increasing SII and TMAO levels with decreasing bone mass, specifically in the population with VFA ≥ 100 cm^2^; However, no such trends were observed in the group with VFA < 100 cm^2^. These findings suggest a potential link between inflammatory state, gut microbiota-related metabolic disorders, and loss of bone mass in centrally obese individuals. Additionally, previous studies have indicated the predictive value of SII for low BMD or OP in postmenopausal women aged over 50 (13). Moreover, recent research has implicated TMAO, a metabolite generated by the gut microbiome, has been associated with OP via the “bone-gut” axis. Fundamental research has revealed that TMAO enhances the expression of peroxisome proliferator-activated receptor gamma and CCAAT/enhancer-binding protein alpha proteins while reducing expression of runt-related transcription factor 2 and Osteopontin proteins through activation of nuclear factor kappa B signaling pathway. Ultimately, this leads to adipogenic differentiation of bone marrow mesenchymal stem cells while inhibiting osteogenic differentiation, resulting in OP (38). Furthermore, TMAO has been shown to enhance TRAP-positive osteoclastogenesis dose-dependently while promoting bone resorption through upregulation of related genes (39).Therefore, in addition to monitoring conventional SII markers, it may be advantageous to include serum TMAO levels as a potential indicator of bone loss in individuals with central obesity.

The study was then conducted on a group of patients with central obesity whose VFA was ≥ 100 cm^2^. The scatter plot revealed a downward trend in WB BMD, LS BMD, and FN BMD as the levels of SII and TMAO increased. Linear model regression analysis demonstrated that even after adjusting for confounding factors such as gender, age, and BMI, SII and TMAO remained negatively correlated with WB BMD, LS BMD, and FN BMD. Binary logistic regression model analysis indicated a positive correlation between levels of SII and TMAO with the incidence of fragility fractures in central obesity patients (Models 1-4). Due to the different impact of obesity on the risk of fractures in various body parts (40), we conducted a statistical analysis on the number of fractures among centrally obese patients, grouped according to different fracture sites. We found no significant differences in SII and TMAO among the groups (data not shown). This suggests that SII and TMAO may not reflect the differences between different fracture sites after a fracture occurs. This may be because SII and TMAO are systemic indicators mainly used to assess overall inflammation and metabolic status, which are not sensitive enough to capture local inflammation or metabolic changes after fractures. Additionally, chronic inflammation and metabolic disorders in centrally obese patients may have already reached a higher baseline level before the occurrence of fractures, resulting in less noticeable fluctuations in SII and TMAO levels after fractures. Furthermore, multivariate logistic regression analysis identified SII (OR = 1.005, 95% CI = 1.003-1.007, *p* < 0.001) and TMAO (OR = 2.733, 95% CI = 1.947-3.330, *p* < 0.001) as independent risk factors for OP in central obesity patients after excluding confounding factors. Additionally, the study evaluated the diagnostic efficacy of SII and TMAO alone as well as their combined detection, with results showing that the AUC of combined detection was the largest, and had a sensitivity reaching up to 94.7%, and specificity at 96.5%, indicating good diagnostic efficacy when used together.

This study is the first to apply VFA grouping and reveal differences in SII and TMAO levels, particularly in central obesity patients with VFA ≥ 100 cm², where elevated SII and TMAO levels were associated with OP, providing preliminary evidence for their potential role in bone metabolism. Although the specific clinical significance of SII and TMAO remains to be further explored, they may serve as potential biomarkers for OP risk screening. We acknowledge that the retrospective analysis of this study cannot establish a causal relationship between SII, TMAO and OP. In the future, we plan to conduct multicenter, large-scale clinical studies to validate the association between SII, TMAO and OP, as well as perform animal intervention experiments to further verify their causal relationship. Additionally, there are several limitations to this study. Firstly, we only considered the patients’ BMD and history of fragility fractures without assessing bone microstructure indicators, thus limiting our comprehensive evaluation of OP. Future studies should incorporate trabecular bone score, high-resolution peripheral quantitative computed tomography, and other indicators to enhance assessment. Secondly, we measured VFA using bioelectrical impedance analysis (BIA) instead of computed tomography (CT), which is the gold standard for VFA measurement. Nonetheless, BIA is a more widely used, cost-effective, and radiation-free method compared to CT. Moreover there exists a significant correlation between VFA measurements obtained via CT and those estimated by BIA in healthy volunteers (41).

## 5 Conclusions

In conclusion, this study revealed significant variations in SII and TMAO levels among overweight and obese patients with different distributions of visceral adipose tissue, which were also found to be correlated with BMD. The peripheral blood levels of SII and TMAO showed an increase as BMD decreased, particularly in the group with VFA ≥ 100 cm². Furthermore, SII and TMAO were significantly associated with BMD and the occurrence of fragility fractures, serving as independent risk factors for the development of OP in individuals with central obesity. The combined assessment of SII and TMAO demonstrated superior diagnostic efficacy.

## Data Availability

The raw data supporting the conclusions of this article will be made available by the authors, without undue reservation.
